# Role of GLI Transcription Factors in Pathogenesis and Their Potential as New Therapeutic Targets

**DOI:** 10.3390/ijms19092562

**Published:** 2018-08-29

**Authors:** Maja Sabol, Diana Trnski, Vesna Musani, Petar Ozretić, Sonja Levanat

**Affiliations:** Laboratory for Hereditary Cancer, Division of Molecular Medicine, Ruđer Bošković Institute, Bijenička cesta 54, 10000 Zagreb, Croatia; maja.sabol@irb.hr (M.S.); diana.trnski@irb.hr (D.T.); vmusani@irb.hr (V.M.); pozretic@irb.hr (P.O.)

**Keywords:** HH-GLI signaling pathway, GLI transcription factors, signal transduction, malformations, cancer, therapy

## Abstract

GLI transcription factors have important roles in intracellular signaling cascade, acting as the main mediators of the HH-GLI signaling pathway. This is one of the major developmental pathways, regulated both canonically and non-canonically. Deregulation of the pathway during development leads to a number of developmental malformations, depending on the deregulated pathway component. The HH-GLI pathway is mostly inactive in the adult organism but retains its function in stem cells. Aberrant activation in adult cells leads to carcinogenesis through overactivation of several tightly regulated cellular processes such as proliferation, angiogenesis, EMT. Targeting GLI transcription factors has recently become a major focus of potential therapeutic protocols.

## 1. Introduction

The Hedgehog-GLI (HH-GLI) pathway is one of the major developmental pathways. Depending on the inputs, the pathway can be directly or indirectly modulated by proliferative and oncogenic signals. The GLI transcription factors have an important role in the intracellular signaling cascade, acting as main mediators of HH-GLI signaling. Considering the complexity of various aspects of the HH-GLI pathway, including crosstalk with other pathways, levels and limitations of Smoothened (SMO) antagonists, the investigators have moved their attention to downstream targets and components. Today, focus has turned to GLI proteins as more attractive potential targets, taking into account both canonical and non-canonical pathway activation by multiple important signaling pathways.

## 2. The HH-GLI Signaling Pathway

The Hedgehog-GLI (HH-GLI) signaling pathway is a conserved pathway that was first discovered in genetic analyses of segmental development in *Drosophila melanogaster* during the 1980s [1]. This pathway plays a critical role during embryonic development, and later in the adult organism in the maintenance of stem cells, tissue homeostasis, hematopoiesis and in the immune system [2,3,4,5,6,7,8]. The pathway has also been implicated in the development of various cancer types [9].

Even though the pathway is conserved in animals, a unique characteristic of vertebrate HH-GLI signaling is the requirement of primary cilia for proper signal transduction [10]. The primary cilium is a long, non-motile, thin organelle protruding from the apical surface of almost all cell types. It is fundamentally important for normal cell signaling during development and homeostasis. These signaling functions are carried out by various signaling molecules localized to the cilium, such as transmembrane receptors that allow for responses to external stimuli and regulatory proteins at the basal body, transition zone and distal regions of the cilium that control the signaling steps [11].

### 2.1. Signaling at the Membrane

In mammals the HH-GLI signaling pathway consists of three HH ligands (Desert, Indian and Sonic Hedgehog, DHH, IHH and SHH), two 12-pass transmembrane receptors named Patched1 (PTCH1) and Patched2 (PTCH2), a 7-pass transmembrane G-protein coupled receptor-like protein Smoothened (SMO) and three transcription factors, named GLI1, GLI2 and GLI3, which are regulated by Suppressor of Fused (SUFU). However, PTCH1 is the main receptor, and PTCH2 is rarely described. In the inactive state, PTCH1, a constitutive inhibitor of HH-GLI signaling, is located in the cilium membrane. This inhibition is carried out by repression of the co-receptor SMO. PTCH1 represses ciliary localization of SMO, a necessary step for pathway activation in mammals [12]. 

**Figure 1:**
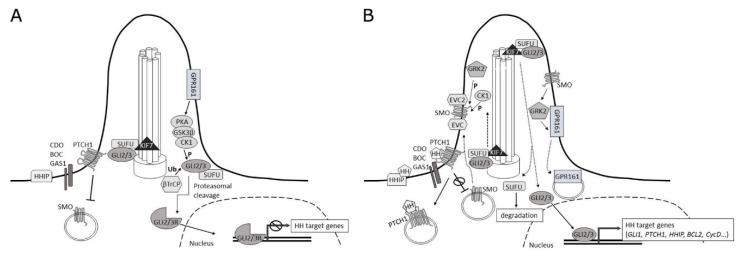
HH-GLI signaling pathway. (**A**) pathway inactive. (**B**) pathway active.

Binding of the ligand HH to PTCH1 relieves the repression of SMO and leads to the activation of the transcriptional factors GLI. GLI1 functions as a transcriptional activator, while GLI2 and GLI3 can act as activators or repressors depending on the cellular context [13]. GLI2 and GLI3 are the primary mediators of signal transduction and *GLI1* being a target gene acts as a positive feedback to reinforce GLI activity [14] (Figure 1).

For high-affinity HH binding PTCH1 requires several co-receptors, namely CAM-related/downregulated by oncogenes (CDO) and brother of CDO (BOC), as well as growth-arrest specific 1 (GAS1). By binding with PTCH1 these proteins form a multimolecular complex and act collectively as co-receptors for HH and to promote signal transduction. Deletion of all three co-receptors results in the near complete abrogation of HH signaling and failure to promote tumorigenesis [15]. The HH proteins are also regulated by glypicans, which enhance their stability and promote their internalization with PTCH1 [16]. Hedgehog interacting protein (HHIP), another cell surface protein has been identified to regulate HH binding to PTCH1 [17]. The general hypothesis is that HHIP acts to bind and sequester HH, making it unavailable to PTCH1 for pathway activation. This affects both the HH secreting cell and the surrounding cells. This makes HHIP a negative regulator of the pathway [18].

In recent years much effort has been put into deciphering of how exactly PTCH1 regulates SMO and how SMO relays the signal to the cytoplasm. Even though there have been numerous discoveries, these questions are still not completely answered. It has been postulated that since PTCH1 harbors homology with the resistance-nodulation-division (RND) family of bacterial membrane transporters, it could serve as a transporter for an endogenous small-molecule SMO modulator [19]. However, a transporter function for PTCH1 has not yet been demonstrated. Cholesterol has been suggested as an endogenous SMO regulator [20,21,22,23,24]. SMO has two separate domains capable of binding steroidal ligands, the extracellular cysteine rich domain (CRD) and the extracellular part of the 7-trans-membrane domain (7TMD), that also binds cyclopamine [25]. It has been found that cholesterol is both necessary and sufficient to activate SMO, but it is unclear which domain is crucial for the activating effect of cholesterol [22,25]. Mutational analyses conducted by Luchetti et al. indicate that cholesterol mediates its activating effect on SMO through the CRD binding-site and which then activates the downstream signaling cascade [25]. It is hypothesized that PTCH1 might act to prevent the access of SMO to cholesterol, or in case that cholesterol functions as a constitutive co-factor of SMO activity PTCH1 might regulate another lipidic inhibitor [24]. Recently Huang et al. have been able to describe the structural basis of SMO activation and have observed that SMO activation leads to the opening of a tunnel that runs from the inner leaflet of the membrane through the 7TM site to an extracellular opening near the CRD. The role of this tunnel is yet to be deciphered, but they propose the tunnel serves as a path for cholesterol involved in SMO activation, to ascend from the membrane to the CRD, providing an answer to the question of how cholesterol might be presented to the CRD in vivo [23]. Another interesting domain is the cytoplasmic tail of SMO, or the C-terminal tail (CTT), which also appears to be involved in the endogenous modulation of SMO through phosphatidylinositol 4-phosphate (PI4P) that promotes its phosphorylation and activation [26].

It has been shown that upon pathway activation, the SMO CTT becomes phosphorylated by casein kinase 1,α (CK1α) and the GPCR kinase 2 (GRK2). These phosphorylation events trigger SMO ciliary localization and induce a conformational switch leading to dimerization of the activated SMO CTT [27,28].

### 2.2. Cytoplasmic Signaling Cascade

There has been much debate on the mechanism of further signal transduction toward GLI activation. GPR161, a G-protein coupled receptor, has recently come into focus and has been proposed to function downstream of SMO to repress basal HH signaling by promoting production of GLI3 repressor form (GLI3R) [29]. Upon HH stimulation, GRK2 would initiate ciliary clearance of GPR161 and promote downstream signal transduction [30].

A study by Pusapati et al. has established that GRK2/3 is required for HH signaling at a step downstream of SMO, but they suggest a GPR161 dependent function in relation to GLI3R-regulated genes, but a GPR161-independent function for regulating high-level HH responses by GLI2 activator form (GLI2A) [31].

A role for EVC and EVC2 proteins in the transduction of the HH signal from SMO to PKA and SUFU has also been suggested. It was proposed that upon HH induced phosphorylation and ciliary accumulation, SMO interacts with EVC/EVC2, which then leads to the inhibition of GLI3R formation and induction of GLI activator formation [32,33].

SUFU is a major negative regulator of HH-GLI signaling activity that directly binds the GLI proteins in the absence of HH stimulation and thereby prevents their translocation to the nucleus and subsequent pathway activation [34]. By tethering the GLI proteins in the cytoplasm, SUFU promotes their degradation or processing into repressor forms [35].

When the pathway is inactive, the binding of SUFU to GLI2 and GLI3 sequesters them in a cytoplasmic complex containing KIF7, a cilium associated kinesin. This prevents full length GLI to enter the nucleus and activate target gene transcription. Also, by masking the GLI NLS (nuclear localization signal) SUFU prevents nuclear accumulation of GLI2 and GLI3 mediated by Kapβ2 [36,37].

Moreover, binding of SUFU to GLI2/3 potentiates the formation of GLI2/3 repressor forms (GLI2R and GLI3R), after sequential phosphorylation of full length GLI by protein kinase A (PKA), glycogen synthase kinase 3 beta (GSK3B) and casein kinase 1 (CK1) within the complex. After phosphorylation, full length GLI2 and GLI3 are cleaved through the ubiquitin-proteasome pathway mediated by the ubiquitin E3 ligase SCFβTrCP, resulting in the formation of N-terminal repressor forms [38,39]. These truncated GLI proteins inhibit target gene transcription by competing with GLI2/3 full length forms or recruit co-repressors [40,41].

Upon signaling activation, the complex comprising of SUFU and GLI2/3 is translocated to the tip of the primary cilium, where its dissociation is initiated. Full length GLI2 and GLI3 are free to translocate to the nucleus as transcriptional activators, while SUFU is degraded. A key function of SUFU is also preserving a pool of full length GLI2/3 proteins by preventing complete degradation mediated by SPOP, and as soon as HH-GLI signaling is activated these proteins are derepressed and readily activate target gene transcription [42,43]. In addition to the cytoplasm, SUFU also regulates GLI proteins in the nucleus, where it is able to recruit specific co-repressor complexes, such as SAP18-mSin3-HDAC and p66β and thereby suppress GLI transcription activity [44,45,46].

### 2.3. Nuclear Signaling

GLI proteins are members of the Kruppel family of transcription factors. They have five conserved zinc-finger DNA binding domains and bind to the consensus sequence GACCACCCA in the promoter of their target genes [47]. GLI proteins can recognize variant binding sites with lower affinity that can still lead to strong transactivation [48]. The outcome of HH-GLI signaling depends on the receiving cell type, and regulates various cellular responses such as proliferation and differentiation (Cyclin D1/2, Cyclin E, E2F1, N-MYC, FOXM1, PDGFRα), cell survival (BCL2), self-renewal (SOX2, NANOG, BMI1), angiogenesis (VEGF, CYR61, ANG1/2), epithelial-mesenchymal transition (EMT) (SNAIL1, ZEB1, ZEB2, TWIST2, MMP9), invasiveness (Osteopontin) and HH-GLI pathway autoregulation (GLI1, PTCH1, HHIP1). Targeting GLI1 leads to amplification of the signal upon pathway activation, while PTCH1 and HHIP1, both negative regulators of the pathway, represent negative feedback loops controlling the extent of HH signaling [49,50].

### 2.4. The GLI Code

The GLI proteins possess both activator and repressor capabilities. GLI2 and GLI3 harbor a repressor domain at their amino terminus and an activator domain at their carboxy terminus. GLI1 on the other hand, lacks the amino-terminal repressor domain and acts as a pathway activator. The combinatorial and cooperative function of the three GLI transcription factors is known as the GLI code [51,52,53]. In the absence of HH signals, the GLI code is driven toward a repressive output (GLIR) leading to pathway downregulation. In this scenario, GLI2/3 are processed into transcriptional repressors and GLI1 is not transcribed. Canonical activation of the pathway through HH blocks GLI processing and promotes target gene activation through active GLI forms (GLIA). Even though at first the regulation of the GLI code seemed to be entirely dependent on the canonical HH-GLI signaling, it has become evident that non-HH signals can also contribute to the regulation of the GLI code, leading to non-canonical HH pathway activation [53]. These new findings contribute to the understanding of how the GLI code is disrupted in human cancer.

## 3. GLI Genes and Protein Isoforms

*GLI1* gene is located at 12q13.3-14.1, the gene is comprised of 12 coding exons and contains 3318 base pairs giving rise to a full length 1106-amino acid protein [54]. *GLI2* gene is located at 2q14, consists of 13 coding exons and contains 4758 bp giving rise to the full length 1586-aa protein [55,56]. *GLI3* gene is located at 7p13, consists of 14 coding exons and contains 4740 bp giving rise to the full length 1580-aa protein [57,58].

**Figure 2:**
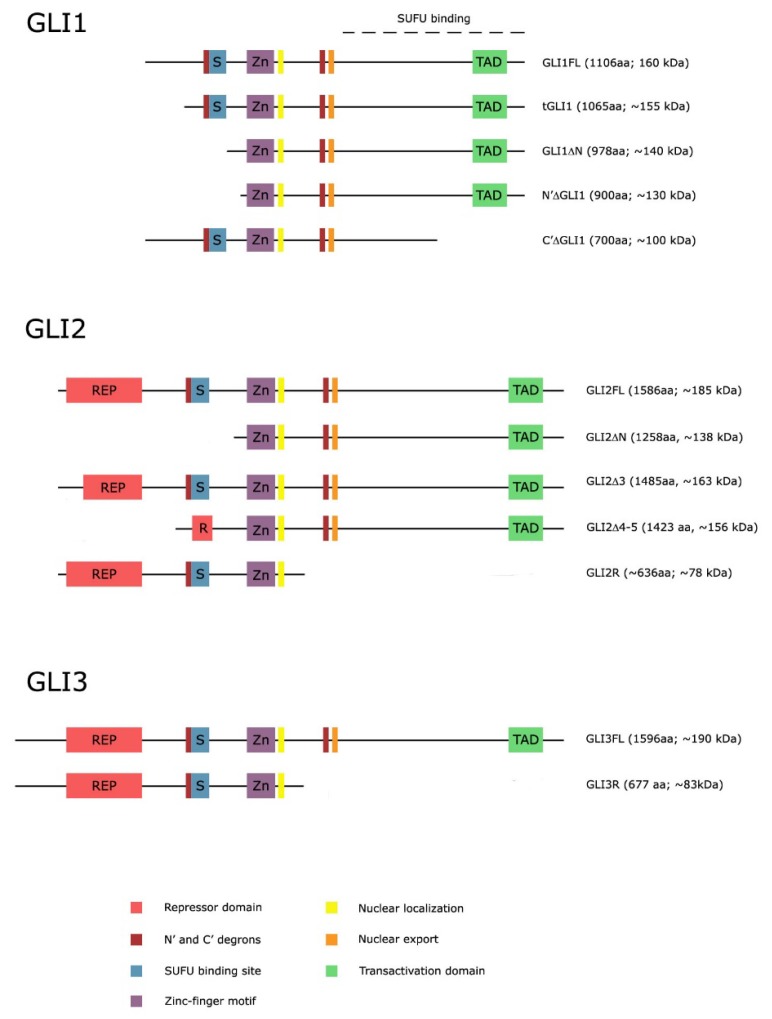
Schematic representation of GLI isoforms.

Recent evidence indicates that both *GLI1* and *GLI2* can undergo alternative splicing, that leads to the synthesis of their truncated variants (Figure 2). GLI1 has currently five known isoforms. Of those five, two are splice variants of the full-length mRNA, and at least one isoform is generated by a posttranslational N-terminal truncation of the full-length protein [59]. The GLI1ΔN splice variant lacks 128 amino acids at the N-terminus due to skipping exons 2 and 3. Even though the GLI1ΔN variant lacks the N-terminal SUFU binding domain, it was shown to be a less potent transcriptional activator than the full-length protein with a weakened ability to translocate to the nucleus. Also, even though GLI1ΔN and GLI1FL have the same DNA binding domain, it seems that there are differences in their target gene activation mechanism [60].

Another splice variant of *GLI1* was identified in malignant glioma. This variant, named tGLI1, lacks the entire exon 3 and part of exon 4 of the full-length *GLI1* gene [61]. This variant has all functional domains present in GLI1 preserved and thus retains the ability to translocate to the nucleus, activate target genes and respond to SHH stimulation. The expression of tGLI1 was detected only in cell lines and primary specimens derived from human glioblastoma and breast cancer, but not normal brain and breast tissue. This is a distinct feature compared to GLI1 and GLI1ΔN as both those proteins are present in normal and cancer cells. In addition, tGLI1 also has multiple direct transcriptional targets that differ from those of GLI1, such as *CD24*, *VEGFA*, *MMP2* and *MMP9*, that can contribute to an aggressive cell phenotype [54,62].

The precise function of tGLI1 compared to GLI1 will need to be investigated in detail. The difference between tGLI1 and GLI1 is 41 amino acids, which is approximately 4.5 kDa. Because of this minimal difference in size, and the fact that most commercial antibodies detect both isoforms, it is possible that in the past some functions correlated with GLI1 have actually been due to tGLI1 [54].

Two isoforms N′Δ GLI1 and the C′Δ GLI1 have been identified as weak activators and repressors, respectively [63]. N′Δ GLI1 lacks the N-terminus of full length GLI1 and since it is produced from the full-length cDNA it is different from the splice variant GLI1ΔN. As opposed to the splice variant, which is exceedingly rare, the N′Δ GLI1 isoform is 10-fold more abundant than the full-length protein, due to loss of a degron N and a key binding site for SUFU. p53 might be involved in N′Δ GLI1 phosphorylation and subsequent inactivation. C′Δ GLI1 on the other hand, might act as a negative feedback as it resembles the GLI3 repressor form, but unlike GLI3R, it is not inhibited by HH signaling [64].

GLI2, like GLI1, possesses splice variants. The N-terminally truncated GLI2ΔN splice variant was at first thought to represent the entire GLI2 protein, but Roessler et al. showed that the full-length GLI2 contains an additional 328 amino acids at the amino terminus, containing the repressor domain. The full length GLI2 was shown to be up to 30-fold less potent than the GLI2ΔN variant [65].

Later on, two more splice variants of GLI2 were discovered, namely GLI2Δ3, a variant arising from skipping exon 3, and GLI2Δ4–5, a variant lacking exon 4 and 5, supporting the hypothesis that alternative splicing may be another important regulatory mechanism for the modulation of repressor and activator properties of GLI2 protein in addition to proteolytic processing [55]. For GLI3, on the other hand, no splice variants have been found and verified to date. 

## 4. Role of GLI in Development

HH-GLI signaling plays a key role during development of both vertebrates and invertebrates, especially during patterning and morphogenesis. The HH proteins are morphogens that induce distinct cell fates in a dose-dependent manner. They can also regulate cell proliferation and control the form of a developing organ. HH-GLI signaling is involved in development of almost every organ during the development of vertebrate embryo (e.g., bone and cartilage, limbs, neurons, cerebellum, eye, gut, gonads, heart, lung, pancreas, muscle, lateral symmetry, angiogenesis) [66]. Therefore, it is not surprising that the defects in HH-GLI signaling lead to development of many different congenital malformations and syndromes.

SHH is expressed in numerous tissues with polarizing activities during development. SHH expression can first be detected in the node, then in the notochord, floor plate of the neural tube, and in the zone of polarizing activity (ZPA) in the limb buds. Teeth, skin and several other tissues with inductive interactions also express SHH during development. The expression of HH-GLI target genes, *PTCH1* and *GLI1*, are mostly restricted to proliferating cells adjacent to SHH-expressing tissues, while *GLI2* and *GLI3* show broader expression in proliferating cells in regions more distant to SHH [67].

Expression of *Gli* genes in different embryonic tissues seem to follow similar rules. For example: during the mouse limb development, at 9.5 days postcoitum (dpc) *Gli2* and *Gli3* are expressed in the entire anterior-posterior (A/P) axis of the newly formed limb bud while *Gli1* is confined to the posterior half. At 10.5 dpc *Gli2* and *Gli3* remain confined to the anterior limb bud while their expression is excluded from the posterior part, while *Gli1* fades from the most posterior domain. At 11.5 dpc the posterior area which corresponds to the ZPA is devoid of any *Gli* expression and is surrounded by a *Gli1*-positive, but *Gli3*-negative domain. The anterior part of the limb bud expresses *Gli2* and *Gli3*. At 12.5 dpc *Gli1* transcripts are found in the condensing mesenchyme while the surrounding mesenchyme expresses *Gli2* and *Gli3* [68].

Mouse *Gli* mutants were generated to discriminate between overlapping and unique requirements for the three genes. *Gli1^−/−^* mutant seem to be normal, fertile and can live past 1 year of age and show no obvious behavioral abnormalities [69]. Mouse *Gli1^−/−^* mutants show changes in T cell development in the thymus [70]. *Gli2* mutants have numerous skeletal defects and abnormal lungs. They also lack a floorplate and adjacent ventral intermediate region (VIR) cells in the spinal cord [69]. Mouse *Gli2^−/−^* show changes in T cell development [71]. *Gli2^−/−^* mouse embryos exhibit severe defects in hindbrain ventral patterning, and it is thought that GLI2 activator function is required for the development of specific progenitor cells that will become motoneurons and interneurons in the ventral neural tube [72]. *Gli2* mutant mice embryos also show abnormal pituitary development, with normally patterned but smaller pituitary, and have decreased number of anterior pituitary secreting hormone cells: specifically, corticotropes, somatotropes and lactotropes [73]. *Gli3^−/−^* mutant mice die around birth, exhibiting severe craniofacial abnormalities, often combined with extracephaly and an enhanced polydactyly with up to nine digits. Mouse *Gli3* mutants show changes in T cell development in the thymus and B cell development in the fetal liver [74,75,76]. Heterozygous mutants show preaxial polydactyly [77].

## 5. Role of GLI in Congenital Malformations and Syndromes

Mutations in *GLI* genes are found in diverse developmental malformations, both syndromic and not. Polydactyly is the only congenital malformation that results from mutations of all three *GLI* genes. Syndactyly and polydactyly—respectively characterized by fused and supernumerary digits—are among the most common congenital limb malformations, with syndactyly presenting at an estimated incidence of 1 in 2000–3000 live births and polydactyly at a frequency of 1 in approximately 700–1000 live births [78]. Polydactyly can most easily be classified as preaxial in which the extra digit is located outside the thumb or great toe; postaxial in which the extra digit is located outside the fifth digit, and central (mesoaxial) polydactyly in which the extra digit is located among three central digits [79].

### 5.1. GLI1

Until recently no *GLI1* mutations have been described in any developmental syndrome. In 2017 Palencia-Campos et al. described three independent families with *GLI1* mutations with apparent recessive mode of inheritance. The phenotype of affected individuals closely resembled Ellis–van Creveld syndrome usually caused by mutations in *EVC1* or *EVC2* genes, normally located in primary cilia. The phenotype includes short stature, bilateral postaxial hexadactyly of hands and feet in two families and postaxial polydactyly type A of feet in the third family with normal stature. Additional features included shortening of distal lower extremities, mild nail dysplasia, atrial septal defect, genu valgum, and high palate. The *GLI1* mutations were found in homozygous form in all more severely affected individuals, but some individuals with polydactyly had the mutation in heterozygous form. Two of the mutations are located at the C-terminus of *GLI1* gene, c.1930C > T and c.2340G > A and are coding for the truncated forms of the GLI1 protein lead to the phenotype with short stature, while the third (c.337C > T) located in the N-terminal part is believed to be subjected to nonsense medicated decay [80].

### 5.2. GLI2

Holoprosencephaly (HPE) is a developmental defect of the forebrain characterized by the incomplete separation of the cerebral hemispheres into distinct halves, followed by facial anomalies in most cases. Clinically, the range of symptoms is very wide, from minor facial signs to complex craniofacial anomalies [81]. Mutations have been found in several genes: *SHH*, *GLI2*, *PTCH1*, *TGIF1*, *ZIC2* and *SIX3*, but also in *GAS1*, *EYA4*, *DISP1*, *TDGF1*, *FOXA2*, *LRP2*, *LSS*, *HHIP*, *SMO*, *BMP4*, *CDON*, *CDC42*, *ACVR2A*, *OTX2*, and *WIF1* [82].

*GLI2* mutations have been reported in patients with HPE and/or HPE-like (HPE-L) phenotypes, and usually associated with pituitary anomalies and postaxial polydactyly [73,83,84]. However, other wide phenotypic variety of other craniofacial anomalies have also been reported [82,85].

Truncating mutations of *GLI2* are usually associated with pituitary anomalies, polydactyly and subtle facial features, not typical HPE [73]. The location of the mutations did not correlate with the clinical findings. Missense mutations are often found in patients with craniofacial defects [82,85].

Several large deletions of *GLI2* have been described in individuals with polydactyly and/or pituitary anomalies. Gustavsson et al. reported a patient with a 2;20 translocation and 20 Mb deletion on chromosome 2q14.2–q22.1, including 43 genes, one of which was *GLI2*. Detected symptoms included hypospadias, postaxial polydactyly, double-left-sided ureters, undescended testes, exotropia and amblyopia of the left eye, growth hormone deficiency, as well as Lupus anticoagulantia [86]. Kevelam et al. reported a 1.3 Mb submicroscopic heterozygous deletion in 2q14.2, which includes *GLI2* and four other genes. The patient had normal psychomotor development, but presented bilateral cleft lip and palate and abnormal pituitary gland formation [87]. Greally et al. reported a patient with an 18.9 Mb deletion containing 54 OMIM genes, including *GLI2*. At birth the patient had congenital right talipes equiovarus, a skin tag on the left fifth finger, a wide anterior fontanelle and dysmorphic ears. Subsequent physical examinations showed changes in the shape of forehead, nose, teeth, eyes and eyebrows, changes on the palms and hands, and mild penile phimosis [88]. Ma et al. reported a patient with 10.79 Mb deletion at 2q13q14.2 which contains 38 OMIM genes. The patient showed hypothyroidism associated with a normal size and position of the thyroid gland, as well as negative thyroid antibodies, global development delay and Mullerian agenesis [89]. Kordaß et al. reported a family with 4.3 Mb deletion at 2q14. At birth the patient had short stature, low weight, microcephaly, ventricular septal defect (VSD) and atrial septal defect type II (ASDII). At age 6 she underwent endocrine evaluation and IGF1 and IGFBP3 showed reduced levels. At age 25 she was 150 cm tall and showed scoliosis, mild facial dysmorphism and abnormal temporal myelinization but psychomotoric development was apparently normal. The mutation was shared with her paternal grandmother who also had small stature [90]. Goumy et al. reported a family with 5.8 Mb deletion at 2q14.1q14.3 encoding 24 genes. The mutation was discovered during prenatal diagnosis due to intrauterine growth retardation and partial agenesis of corpus callosum. The patient was born with severe growth retardation. At 6 months, he demonstrated a wide range of relatively mild altered facial features. At 9 months, he showed mild developmental delay. His mother and one brother had the same mutation. The mother had learning difficulties at school, but no other phenotypic abnormalities. The brother had similar facial features as the proband as well as psychomotor retardation [91]. Niida et al. reported a patient with 6.6 MB deletion at 2q14.1q14.3 encoding 28 OMIM genes. The patient had cleft palate and flat nasal bridge, but no polydactyly or other facial or exterior dysmorphic features. She had learning difficulties in school, social communication problems and was diagnosed with selective mutism. She had short stature and delayed puberty [92].

### 5.3. GLI3

Greig cephalopolysyndactyly syndrome (GCPS) is a congenital pleiotropic syndrome associated with multiple anomalies. It is rare, with estimated range of 1–9/1,000,000. It is caused by the loss of function of *GLI3* gene and is inherited in an autosomal dominant pattern [93]. Individuals with large deletions encompassing *GLI3* show more severe phenotype [57,92]. Mutations in the 5′ region close to the or within the zinc finger domain cause the majority of GCPS [94], other mutations have been detected, such as missense, splicing or truncating mutations elsewhere in the gene. They lead to haploinsufficiency, loss of the DNA-binding capacity, or activation of nonsense-mediated mRNA decay, or formation of unstable or mislocalized proteins [57] (Table 1).

Acrocallosal syndrome (ACS, ACLS) was first described in 1979 and has a lot of the same features of GCPS, but usually with a more severe phenotype. It is very rare and only about 50 cases have been reported. The disorder is genetically heterogeneous and in most cases the etiology is unknown, although autosomal recessive mutations in *KIF7* gene and heterozygous missense mutations in *GLI3* have been reported [95].

Pallister-Hall syndrome (PHS) displays a wide range of severity, from very mild cases with subtle insertional polydactyly to lethal cases [57]. It is inherited in an autosomal dominant pattern and shows a complete penetrance. It is a rare syndrome and the prevalence is unknown [97]. It is caused mainly by truncating mutations in the middle third of the *GLI3* gene, resulting in a constitutive repressor protein [57].

## 6. Activation of GLI in Tumorigenesis

Modes of HH-GLI signaling activation are different among different cancer types. Some tumor types depend on mutations of specific pathway components such as PTCH1, SMO and SUFU. This is mostly the case in basal cell carcinoma and medulloblastoma [98,99]. Another example are tumors that depend on specific gene amplification, more precisely on *GLI1* gene amplification, such as glioblastoma or rhabdomyosarcoma [54,100].

HH-GLI signaling pathway or more precisely, GLI proteins activate the transcription of many target genes which are involved in various aspects of tumorigenesis [49]. For example, cell proliferation is enhanced by expression of D-type cyclins [101]; production of B-cell lymphoma 2 (BCL2), an anti-apoptotic protein, mediates cell survival [102]; angiogenesis is activated by upregulation of angiopoietins and vascular endothelial growth factor (VEGF) [103]; transcription of SNAG family of transcriptional repressors initiates the epithelial-to-mesenchymal transition in metastasis [104], while GLI-mediated regulation of SOX2 and NANOG expression facilitates self-renewal of stem cells [105,106]. In consequence, it is expected that dysregulation of HH-GLI signaling could lead to wide variety of malignancies [107].

Aberrant HH-GLI pathway activation, usually manifested by upregulation of GLI1 and PTCH1 expression, has been found in a multitude of human cancer types, including basal cell carcinoma (BCC), medulloblastoma (MB), glioma, melanoma, gastrointestinal, colon, breast, lung, liver, prostate and pancreatic cancer [108]. This aberrant activation can be ligand dependent, ligand independent or caused by genetic and epigenetics mechanisms. The latter include inactivating loss-of-function mutations and promoter hypermethylation/transcriptional inactivation of negative regulators of the pathway (e.g., PTCH1, SUFU) or activating, gain-of-function mutations and promoter hypomethylation/transcriptional activation of positive regulators of the pathway (e.g., SHH, SMO) [109].

### 6.1. Ligand Dependent Signal Transduction

Ligand-dependent activation of the pathway can be autocrine, paracrine or reverse paracrine. In the autocrine model tumor cells secrete and respond to HH ligands and show increased ligand expression in the absence of genetic mutations of HH-GLI components. This type of signaling has been identified in lung, pancreatic, gastrointestinal, colon and prostate cancer as well as glioma and melanoma [110,111,112,113,114,115,116,117,118,119]. In the paracrine activation of HH-GLI signaling, tumor cells produce the ligand HH, which then activates HH-GLI signaling in the surrounding stromal cells. Activation of the pathway in these cells leads to production of factors that sustain tumor cell growth. This type of signaling has been identified in experimental models for prostate, pancreatic and colon cancer [120,121]. In the reverse paracrine model, the ligand is produced by stromal cells, which activates the signaling pathway in tumor cells. This type of signaling activation has been described in B-cell lymphoma, multiple myeloma and leukemia [107]. The HH-GLI signaling pathway has also been implicated cancer stem cell (CSC) self-renewal, contributing to increasing tumor initiating cell populations, growth and tumorigenicity. Activation of HH-GLI signaling has been detected in CSCs of glioma, pancreatic cancer, breast cancer, melanoma, colon cancer, as well as some hematological malignancies [107,122,123].

### 6.2. Ligand Independent Non-Canonical Activation and Cross-Talk with other Pathways

In addition to ligand-dependent, canonical pathway activation, non-canonical activation of the pathway through cross-talk with other oncogenic pathways has been detected in many cancer types. This type of signaling bypasses the ligand-receptor signaling axis and directly relies on activation of the GLI transcription factors through interactions with members of other signaling pathways. It is mainly investigated in the context of malignant diseases in order to understand the underlying mechanisms of GLI activation that could shed light on potential therapeutic strategies.

To date there are multiple lines of evidence that support the cross talk between the HH-GLI signaling pathway and the RAS-RAF-MEK-ERK, PI3K/AKT and TGFB/SMAD signaling pathways. How exactly the RAS-RAF-MEK-ERK cascade causes activation of GLI1 transcription is not completely understood. A study by Whisenant et al. proposes that this signaling axis regulates GLI1 and GLI3 through their phosphorylation by ERK and JNK MAP kinases [124]. It has been shown that oncogenic KRAS can block GLI1 degradation and lead to activation of HH-GLI signaling in pancreatic cancer cells [125]. Also, oncogenic NRAS and HRAS increase GLI1 function in melanoma cells, and HH-GLI signaling is required for NRAS-induced mouse melanoma growth [118]. Riobo et al. have shown that activation of the PI3K/AKT signaling pathway increases SHH-induced transcriptional activity of GLI through antagonizing PKA-dependent GLI2 inactivation, proposing a synergistic role for PI3K/AKT and HH-GLI signaling pathways. Therefore, loss of PTEN, a negative regulator of PI3K, or overexpression of IGF1, which are frequent events in tumors, could upregulate PI3K/AKT activity to stimulate even low levels of HH-GLI signaling [126]. TGFB signaling has been shown to regulate HH-GLI activity independently of SMO by directly promoting GLI2 transcription through SMAD3/β-catenin cooperation [127]. TGFB and RAS signaling regulates the expression of GLI1 and its target genes in pancreatic ductal adenocarcinoma [128]. An interaction between EGFR signaling and the HH-GLI pathway has also been reported. Oncogenic transformation of the cells can be also induced by synergistic activation of the HH-GLI and epidermal growth factor receptor (EGFR) pathways. This activation depends on EGFR-mediated activation of the MEK/ERK signaling which induces transcription factor AP-1 (JUN) that cooperates with GLI1 and GLI2 [129]. Members of the Protein kinase C (PKC) family are also known to modulate HH-GLI signaling. PKCα increases GLI1 transcription in a MEK/ERK dependent manner, while aPKC ι/λ upregulates HH signaling by directly phosphorylating and activating GLI1. Since aPKC ι/λ is also HH target gene, this positive feedback loop causes sustained HH activation. PKCδ, on the other hand prevents GLI1 nuclear localization and transcriptional activity, leading to silencing of HH signaling [130,131]. The TNFα/mTOR signaling pathway can activate HH signaling through phosphorylation of GLI1 protein at Ser84 by S6K1, which leads to its release from SUFU in esophageal, pancreatic and gastrointestinal cancer cells [132]. Pro-inflammatory cytokines like TNF and IL1B can cause GLI1 overexpression by direct binding of NFKB1 transcriptional complex to *GLI1* promoter, what leads to accumulation of GLI1 in the nucleus. This was observed in pancreatic carcinoma [133], while decreased GLI1 expression caused by inhibition of NFKB was observed in breast cancer [134]. The WIP phosphatase has been shown to increase GLI1 function in melanoma by promoting GLI1 nuclear localization, protein stability and transcriptional activity [135]. P53 has also been shown to interact with GLI1 in a negative reciprocal manner. P53 inhibits the activity, nuclear localization and protein levels of GLI1, while GLI1 conversely inhibits p53 through activation of MDM2 [50,64,136,137]. GLI1 is a direct transcriptional target of EWSR1-FLI1 oncoprotein which is a fusion transcript found in Ewing sarcoma family of tumors and consists of *EWSR1* (RNA binding protein 1) and *FLI1* (Fli-1 proto-oncogene, ETS transcription factor) genes [138]. MYC proto-oncogene directly regulates transcription of *GLI1* which has an anti-apoptotic role in Burkitt lymphoma [139]. Atonal bHLH transcription factor 1 (ATOH1) directly regulates transcription of *GLI2* in granule neuron precursors, the cell of origin of medulloblastoma, where it plays a critical role in tumorigenesis [140]. Another developmental pathway aberrantly active in some cancers, the Wnt/β-catenin pathway, shares reciprocal interactions with HH-GLI signaling on level of GLI1 inducing expression of SNAIL or sFRP1 [141]. A recent study by our lab proposed non-canonical activation of GLI3 in colon cancer cells through a novel mechanism involving deregulated of GSK3B [142].

### 6.3. Genetic Changes

For long time *GLI1* gene has been considered as a unique member of the HH-GLI pathway because at that time no somatic mutations have been reported in tumors and it was thought that oncogenic potential of *GLI1* could be manifested primarily through either gene amplification or an alternative splicing when novel truncated *GLI1* splice variant, tGLI1, with a gain-of-function characteristics was discovered in glioblastoma multiforme and breast cancer [54,61].

Additional post-transcriptional mechanism of *GLI1* regulation has been observed on the level of mRNA where nucleotide 2179 is converted to inosine by adenosine deamination (so called A-to-I RNA editing). This leads to a change from arginine to glycine at amino acid position 701 in SUFU binding domain and thus changed GLI1 protein has reduced responsiveness to both negative HH-GLI signaling regulator SUFU and positive regulator DYRK1A kinase. RNA editing of *GLI1* has been detected in fetal and adult brain, lung, colon, pancreas and ovary tissues, while, on the contrary, in corresponding tumor cell lines the extent of editing was significantly decreased [143]. *GLI1* transcript editing rates were observed higher in relapsed multiple myeloma and plasma cell leukemia, leading to increased GLI1 activity and consequently to malignant regeneration in multiple myeloma [144].

#### 6.3.1. Gene Amplification

*GLI1* gene has been originally isolated from human glioma cells where it was found being amplified more than 50-fold [145]. Therefore, a connection of GLI proteins with tumorigenesis could have been observed from the beginnings of HH-GLI pathway research in humans. Amplification of *GLI1* and *GLI2* loci has been detected in medulloblastoma, the most common malignant brain tumor in children [146]. Gain of chromosomal region 12q13.2–q13.3 which surrounds *GLI1* genomic locus has been linked to breast cancer development [147,148]. In addition, amplification of *GLI1* has been also observed in rhabdomyosarcoma [149] and *GLI2* in oral squamous cell carcinoma [150].

#### 6.3.2. Gene Translocation (Fusion)

In several cases of histologically distinctive soft tissue tumors with pericytic phenotype a new mechanism of *GLI1* activation through fusion with actin beta gene (*ACTB*) has been discovered, and this type of tumor has been defined as ‘pericytoma with t(7;12)’ [151]. An identical fusion transcript including the 5′-part of *ACTB* and the 3′-part of *GLI1* has been also observed in one case of primary bone cancer [152].

#### 6.3.3. Short Genetic Variations

Targeted sequencing of *GLI* genes in cancers has been conducted in several studies. Genetic analysis of *GLI1* in Merkel cell carcinoma, an aggressive neuroendocrine skin cancer, has discovered a frequent synonymous substitution c.576G > A found in exon 5 [153]. HH-GLI pathway mutation analysis in T-cell acute lymphoblastic leukemia discovered the presence of two rare somatic missense substitutions—GLI1:c.C1613T (p.S538F) and GLI3:c.G2179A (p.G727R). Both of them are located downstream of the zinc finger domain and considered harmful for the protein by in silico prediction. One additional missense substitution predicted to be damaging has been found GLI3:c.G1097T (p.R366L) but due to the lack of germline DNA it was not possible to determine whether this sequence variation is acquired or inherited [154]. Genetic analysis of frameshift mutation c.821delG in exon 8 of *GLI1* in gastric and colorectal cancers has shown that this mutation is more prevalent in CRC than GC and that all mutations have been found in cancers with high microsatellite instability [155].

With an increased application of massive parallel sequencing of whole cancer genomes, more mutations in *GLI1* and *GLI3* have been discovered in breast, colorectal, pancreatic cancers and soft-tissue sarcomas [156,157,158,159]. The quick overview of the Catalogue of Somatic Mutations in Cancer (COSMIC) database shows that there are about 100, 30 and 60 sequence variants that could a priori be considered as pathogenic (nonsense substitutions and frameshift indels) found in *GLI1*, *GLI2* and *GLI3*, respectively [160]. These short genetic variations have been found by sequencing genomes of wide variety cancer types such as breast, skin, kidney, lung, pancreatic, prostate, intestine cancer, etc. As expected, there are many more discovered missense and synonymous substitutions whose potential functional and clinical impact remains to be verified. One study which has shown how missense substitutions could have impact on tumorigenic potential of GLI1 protein has been conducted by Huntzicker et al. [161]. They demonstrated that degradation of GLI1 is regulated by two independent destruction signals called degron D_C_ and degron D_N_, and that amino acid changes in one or both degrons stabilize GLI1 protein and rapidly accelerates tumor formation in transgenic animals [161].

### 6.4. Epigenetic Changes

Epigenetic changes are heritable phenotype changes without alterations in the DNA sequence. These changes are caused by processes which involve DNA methylation in promoter region of the genes [162,163,164], remodeling of nucleosome by histone modifications [165] and regulation of gene expression by non-coding RNA molecules such as mircoRNAs (miRNAs) and long non-coding RNAs (long ncRNAs) [166]. Dysregulation of all of these processes has been associated with the development of cancer [167].

#### 6.4.1. DNA/Protein/Histone Methylation

*GLI3* overexpression caused by promoter hypomethylation has been observed in gastric cancer [168] but its promoter seems not to be affected by hypermethylation in MB [169]. In breast cancer *GLI1* is upregulated due to the loss of lysine methyltransferases (KMT) SETD7 which can put repressive marks on histones within *GLI1* promoter and loss of these marks results in *GLI1* overexpression [170]. Full-length, but not truncated, GLI3 protein can be methylated by SETD7 at K436 and K595 amino acid residues. This methylation increases stability and DNA-binding ability of GLI3 and consequently enhances the HH-GLI pathway activation, which was demonstrated in tumorigenesis of non-small cell lung cancer [171].

Recently it was discovered a non-coding three-exon RNA transcript positioned head-to-head with *GLI1* gene. This antisense RNA (termed GLI1AS) negatively regulates expression of *GLI1*, which, on the contrary, as well as TGFB, positively regulates GLI1AS expression. GLI1AS causes local alterations of chromatin structure by increasing the silencing mark H3K27me3 and reducing the recruitment of RNA polymerase II to this locus, what leads to decreased *GLI1* transcription. GLI1AS expression showed high positive correlation with *GLI1* expression in basal cell carcinoma and breast cancer [172].

#### 6.4.2. RNA Interference

The expression of miRNAs and their target genes are generally inversely correlated. *GLI1* is regulated by miR-218 and miR-324-5p, which are both lost in MB [173,174,175,176]. In gastric cancer, *GLI1* is upregulated due to the loss of miR-202-3b and miR-133b [177,178]. miR-218 downregulates *GLI2*, but miR-106b, which is overexpressed in MB and BCC, upregulates *GLI2* [174,175,179,180,181,182]. *GLI2* is targeted by miR-326 which is lost in non-small-cell lung cancer [183]. In hepatocellular carcinoma, *GLI2* is upregulated due to the loss of miR-200a [184,185], while *GLI3* due to the loss of miR-378a-3p [186]. *GLI3* is upregulated in BCC because of the miR-378 loss, which controls expression of *GLI3* in healthy tissue [179,186]. In gastric cancer *GLI2* is upregulated due to the upregulation of lncRNA metastasis associated lung adenocarcinoma transcript 1 (MALAT1). MALAT1 downregulates miR-202 which normally targets *GLI2* [187].

## 7. GLI Proteins as Therapeutic Targets

Targeting the HH-GLI signaling pathway is one of the recent approaches in cancer therapy. Originally, the HH-GLI signaling pathway was targeted at the level of SMO, in an attempt to bypass the inactivating PTCH1 and activating SMO mutations often found in Hedgehog-associated tumors like medulloblastoma and basal cell carcinoma. The first inhibitor, cyclopamine, was extracted from the *Veratrum californicum* plant after it was associated with congenital malformations in lambs feeding on this specific flower [188]. Cyclopamine was found to be inappropriate for clinical use due to poor solubility and pharmacokinetic properties [189]. Therefore, several companies started developing SMO inhibitors, and today some of those inhibitors are used in the clinic. GDC-0499, known as vismodegib, is used for treatment of advanced basal cell carcinoma [190,191]. NVP-LDE225 (sonidegib) has also been approved for locally advanced basal cell carcinoma treatment [192], while IPI-926 (saridegib) is still undergoing clinical trials [193]. All three SMO inhibitors are under investigation for treatment of solid tumors [194,195,196], and a known and approved antifungal drug itraconazole is being examined for potential clinical application for treatment of basal cell carcinoma [197]. Unfortunately, resistance to SMO inhibitors has already been reported in patients, with distinct mechanisms that include mutations in SMO, amplification of GLI2 or upregulation of synergistic signaling pathways such as PI3K. Gӧnissen et al. suggest some possible solutions in their review: second-generation SMO inhibitors with a different mechanism of action, HH-GLI pathway inhibitors downstream of SMO, or combination strategies with other signaling pathways [198]. Other HH-GLI inhibitors, targeting components downstream of SMO, have not yet been included in clinical trials.

### 7.1. GLI Inhibitors

Not all tumors with HH-GLI pathway activity show the canonical signal transduction. In those tumors, canonical SMO inhibition is not as effective because GLI proteins are activated regardless of SMO by other signaling cascades. That is the reason that the focus has been shifting from SMO inhibitors to GLI inhibitors. The first and most frequently used GLI inhibitors GANT58 and GANT61 were detected in a cell-based screen by Lauth et al., and GANT61 was shown to be more potent of the two [199]. Their advantage, compared to the SMO inhibitors, is inhibition of both canonical and non-canonical activation of GLI proteins. The same authors have also shown that GANT61 impairs GLI1-DNA binding [199], and this is due to direct binding of GANT61 to GLI1 protein. The binding site is conserved between GLI1 and GLI2 proteins, making GANT61 an inhibitor of both GLI1- and GLI2-mediated transcription [200].

The most commonly reported effect of GANT61 is decreased cell survival through induction of apoptosis. This has been demonstrated in numerous tumor models (Table 2). Other frequently reported effect is the G1/S phase cell cycle arrest [201]. Recently discovered additional effects of GANT61 include the induction of double strand breaks (DSB) [202], generation of reactive oxygen species (ROS) by the mitochondria [203], decreased induction of EMT [204] and induction of autophagy [205]. Treatment with GANT61 affects GLI target gene expression, such as human telomerase reverse transcriptase (*TERT*), additionally decreasing the proliferative potential of cells [206].

When comparing upstream canonical HH-GLI inhibition (SMO inhibition) with downstream inhibition (GLI inhibition), it is evident that downstream inhibition is more effective in tumor therapy, as shown by several authors in recent studies. For example, Benvenuto et al. compared the two types of inhibition on breast cancer growth in a nude mouse xenograft model, and demonstrated that the effect of GANT61 is more pronounced than GDC-0449 [207]. The same effect was demonstrated on a panel of 18 cell lines derived from the most common and aggressive pediatric tumors, where GANT61 inhibition was more effective than SANT1 [208]. Bleomycin-induced lung fibrosis was also found to be more responsive to GLI inhibition than SMO inhibition [209]. In a xenograft model of squamous lung cancer GANT61 was very effective while GDC-0449 resulted in limited cytotoxicity [210]. GANT61 was shown to be more potent that cyclopamine in rhabdomyosarcoma, both in vitro and in vivo [211], in neuroblastoma [201], in biliary tract tumors [212] and in chronic lymphocytic leukemia [213]. GANT61 is more potent than GDC-0449 in prostate cancer cells [214] and acute myeloid leukemia [215].

Compounds affecting protein regulators of GLI activity have also been used (listed in Table 2). For example, HH-GLI pathway inhibitors (HPIs) identified by Hyman et al. act on GLI processing and ciliogenesis [241]. Imiquimod, a known Toll-like receptor 7/8 (TLR7/8) antagonist, acts on protein kinase A (PKA), which regulates GLI processing and phosphorylation. Upon treatment, processing is increased, as is phosphorylation of GLI2 and accumulation of GLI3R [248]. Perifosine, an AKT/PI3K inhibitor, downregulates GLI1 expression through the PI3K/AKT pathway [250]. Nanoquinacrine regulates GSK3B activity, which in turn regulates GLI proteins [251].

Other compounds already used in the clinic have also shown to be useful in GLI inhibition, for example arsenic trioxide (ATO) which is used for treatment of acute promyelocytic leukemia (APL). Yang et al. demonstrated that ATO therapy in APL patients leads to compete remission in 86% of cases, and these patients show a significant downregulation of GLI2 and SMO gene expression, and marginal downregulation of PTCH1 and GLI1 [254]. Beauchamp et al. have demonstrated that ATO treatment affects GLI1 protein activity without modifying its cellular localization, by direct binding to the protein [229]. On the other hand, Kim et al. have shown that ATO inhibits activation of GLI2 by preventing its transport from the primary cilium [228]. Han et al. have proposed that ATO may act as a HH-GLI pathway inhibitor by replacing the zinc ion in the GLI zinc finger proteins, rendering them inactive [227]. Histone deacetylase 6 (HDAC6) inhibitors are novel potential targets in Hedgehog-associated tumors, since HDAC6 can regulate GLI2 and GLI3 protein expression [255]. Even aspirin, a widely used analgesic, antipyretic and anti-inflammatory drug, has shown inhibitory effect on the HH-GLI signaling pathway, and it sensitizes malignant glioma cells to temozolomide therapy [243].

Epigenetic regulation of GLI activity has also been demonstrated. Bromodomain and extra terminal (BET) proteins, or BRD4 more specifically, binds directly to the *GLI1* and *GLI2* promoters and regulates their transcription. BRD4 inhibitors JQ1 and I-BET151 have been shown to inhibit HH-GLI signaling and tumor growth [252,253].

One of the issues in treating solid tumors is delivery to target cells. Nanoparticles are being tested to improve the bioavailability and delivery of specific compounds. For example, glabrescione B was encapsulated into oil-cored polymeric nanocapsules by Ingallina et al, in an attempt to increase delivery of high doses of glabrescione B to solid tumors [256]. HH-GLI pathway inhibitor HPI-1 was encapsulated in nanoparticles from poly (lactic-co-glycolic acid) (PLGA) conjugated with polyethylene glycol (PEG) and successfully used in mouse models of medulloblastoma and pancreatic cancer xenografts [257].

Recently, several groups have started the search for better and novel GLI antagonists, mostly using high-throughput screens of various synthetic or natural compounds, but also virtual libraries. To date, several novel specific GLI antagonists have been identified: glabrescione B [237], physalin H [238], HPI-1 through -4 [241], vitretrifolin D [239], cynanbungeigenin C and D [234], and compound 29a [247].

### 7.2. Combining GLI Inhibitors with other Chemotherapeutic Agents

HH-GLI signaling pathway is a good target for possible combination treatment. Inhibition of this pathway with other oncogenic pathways often gives improved synergistic effects compared to individual therapy. Table 3 shows an overview of several studies examining combined effects of downstream Hedgehog inhibition with inhibition of other pathways, and in the majority of cases the effect is synergistic. It is interesting to note that in breast cancer cells, combined treatment of tamoxifen with SMO inhibitor exerts a different response that combined treatment with GANT61. In the case of cyclopamine, the cells show short-term survival while this is not the case when using GLI inhibitors [258,259].

### 7.3. Role of GLI Proteins in Chemoresistance

GLI protein expression is often associated with resistance to chemotherapeutics, and loss of GLI, either chemically or by knockdown, re-sensitizes the cells to chemotherapy (Table 4). For example, in glioma cells, GLI1 induces non-canonical temozolomide resistance and its knockdown or treatment with aspirin re-sensitizes the cells to the drug [243]. In pancreatic cancer cells GLI1 knockdown, perifosine treatment or GANT61 treatment sensitize the cells to gemcitabine. Perifosine showed the same sensitizing effect to gemcitabine treatment in vivo on pancreatic tumor xenografts [250]. In large cell neuroendocrine carcinoma, downregulation of GLI1/2 sensitizes cells to cisplatin [217].

The mechanism by which GLI proteins impart resistance in still not elucidated. Subpopulations of cells remaining after therapy often show upregulation of the HH-GLI signaling pathway, and these resistant cells repopulate the tumor mass and generate treatment-resistant tumors. Most commonly detected mechanism is the aberrant activation of the ABC transporters, which can be regulated by the HH-GLI signaling pathway [301]. Another proposed mechanisms is induction of the glucuronidation in acute myeloid leukemia (AML), where GLI1 is sufficient to drive UGT1A-dependent glucuronidation of ribavirin and cytarabine, leading to resistance [300]. Resistance to cisplatin is imparted by enhanced DNA repair, and by changing cellular accumulation of the drug through modulation of the c-Jun phosphorylation cascade [302].

## 8. Conclusions

The HH-GLI signaling pathway is a biologically important pathway regulating development, stemness, wound healing and cell survival. It is a tightly regulated system which in the adult organism remains active only in the stem cell population. Major effector of the pathway are the GLI proteins, which can act as activators or repressors depending on the context. Mutations, epigenetic changes or non-canonical activation leading to upregulation of GLI proteins have been associated with numerous congenital malformations and tumors. Therefore, GLI proteins have become attractive molecular targets for novel tumor therapies, since the pathway has been associated with one third of all tumor types. The major problem in tumor therapy today is development of resistance, even to targeted therapies. It is believed that a small subpopulation of tumor cells reacts to therapy by activating numerous signaling pathways in an effort to survive. One of the mechanisms is the activation of HH-GLI signaling pathway, mostly non-canonically through other signals affecting GLI proteins. Consequences of crosstalk between HH-GLI and other pro-tumorigenic pathways include activation of a positive feedback loop, in which activation or accumulation of a component from the first pathway leads to the activation or accumulation of another component, which creates a vicious cycle enhancing cancer progression. For that reason, research focus has shifted toward discovery of successful combined targeted therapies that should be applied in the early stages of disease.

## Figures and Tables

**Table 1:** *GLI3*-associated syndromes and their features.

Syndrome/Condition	MIM ID#	Hand Defects	Feet Defects	Cranial Malformations	Intellectual Disability	Other	Reference
Greig cephalopolysyndactyly syndrome	175700	postaxial polydactyly, cutaneous syndactyly	preaxial polydactyly, cutaneous syndactyly	hypertelorism, macrocephaly with frontal bossing	sometimes	central nervous system (CNS) anomalies, hernias	[93]
Greig cephalopolysyndactyly contiguous gene deletion syndrome	175700	postaxial polydactyly, cutaneous syndactyly	preaxial polydactyly, cutaneous syndactyly	hypertelorism, macrocephaly, hydrocephalus	often	seizures, ophthalmologic findings	[57,92]
Acrocallosal Syndrome	200990	preaxial and postaxial polydactyly, cutaneous syndactyly	preaxial polydactyly	macrocephaly, widely spaced eyes, absence of the corpus callosum, hydrocephalus	yes	seizures, interhemispheric cysts, umbilical hernia	[95,96]
Pallister-Hall syndrome	146510	central polydactyly, postaxial polydactyly	none	bifid epiglottis	no	hypothalamic hamartoma, imperforate anus, renal abnormalities, genital defects	[57,97]

**Table 2:** List of GLI inhibitors and their effects in various tumors.

Inhibitor	Mode of Action	Model/Samples	Effect	Reference
Compounds affecting GLI activity				
GANT58	GLI antagonist	Acute T lymphocytic leukemia	Decreased cell viability, G1/S accumulation	[216]
GANT61	Inhibition of GLI1/DNA and GLI2/DNA binding	Colon carcinoma	Cytotoxicity, induction of DNA damage, G1/S accumulation	[202]
		Large cell neuroendocrine carcinoma of the lung	Inhibition of cell growth, sensitivity to cisplatin	[217]
		Prostate cancer	Decreased cell viability, induction of apoptosis, downregulation of stem cell markers	[214,218]
		Acute myeloid leukemia	Inhibition of cell growth and colony formation, induction of apoptosis	[215,219]
		Rhabdomyosarcoma	Inhibition of xenograft growth, induction of apoptosis, downregulation of EMT markers	[220]
		Neuroblastoma	Decreased cell viability, induction of apoptosis, induction of autophagy, inhibition of xenograft growth	[201,221]
		Ewing’s sarcoma family tumor	Cytotoxicity, induction of apoptosis	[222]
		Ovarian cancer	Reduced migration and invasion, inhibition of xenograft growth	[223]
		Squamous lung cancer	Inhibition of cell growth, induction of apoptosis, inhibition of xenograft growth	[210]
		Pancreatic cancer stem cells	Decreased cell viability, reduced migration, invasion and spheroid formation, induction of apoptosis, inhibition of xenograft growth	[204]
		T-cell lymphoma	Decreased cell viability, induction of apoptosis	[224]
		Mesothelioma	Cytotoxicity, induction of apoptosis, G1 accumulation	[203]
		Biliary tract tumors	Cytotoxicity, induction of apoptosis	[212]
		Cervical cancer	Reduction of proliferation and survival, induction of apoptosis, generation of ROS	[225]
		Melanoma	Decreased cell viability, induction of apoptosis, reduced colony forming	[226]
arsenic trioxide (ATO)	Inhibition of GLI1/GLI2 activity	Pancreatic cancer stem cells	Inhibition of cell growth, induction of apoptosis, reduced migration	[227]
		Medulloblastoma	Cytotoxicity, inhibition of xenograft growth	[228,229]
		Osteosarcoma	Inhibition of cell growth, reduced colony forming, induction of apoptosis, inhibition of xenograft growth	[230,231]
		Prostate	Inhibition of cell growth	[232]
		Rhabdoid tumors	Cytotoxicity, induction of apoptosis, inhibition of xenograft growth	[233]
cynanbungeigenin C and D (CBC and CBD)	Gli1 antagonists	Medulloblastoma	Inhibition of allograft growth	[234]
gedunin	GLI inhibition	Pancreatic cancer	Inhibition of cell growth, induction of apoptosis, reduced migration, downregulation of EMT markers, inhibition of xenograft growth	[235]
GLI-I	GLI inhibitor	Malignant pleural mesothelioma	Cytotoxicity, induction of apoptosis, inhibition of xenograft growth	[236]
glabrescione B	Inhibition of GLI1/DNA binding	Medulloblastoma, basal cell carcinoma	Inhibition of cell growth, reduced spheroid formation, induction of apoptosis, inhibition of allograft growth	[237]
physalin H	GLI1-DNA complex formation inhibition	Pancreatic and prostate cancer	Cytotoxicity	[238]
vitretrifolin D	Inhibition of GLI1-DNA binding	Prostate and pancreatic cancer	Cytotoxicity	[239]
solasonine	GLI-mediated transcription	SHH-LIGHT2 reporter cells	downregulation of GLI1 and PTCH1 expression	[240]
Compounds affecting GLI stability and trafficking				
HPI-1-4	Different effects on GLI stability and trafficking	Breast cancer	Inhibition of cell growth, induction of apoptosis, reduced cancer stem cell population, reduced migration	[241,242]
aspirin	Inhibits GLI1 translocation to nucleus	Glioma	Cytotoxicity, induction of apoptosis, reduced migration and invasion, downregulation of EMT markers,	[243]
Compounds affecting GLI expression				
genistein	GLI1 expression	Gastric cancer	Downregulation of stem cell markers, reduced invasive capacity	[244]
		Breast cancer	Inhibition of cell growth, reduced colony forming, induction of apoptosis, inhibition of xenograft growth	[245]
Quinolone-1-(2H)-ones	GLI expression	SHH-LIGHT2 reporter cells	Downregulation of GLI1 and PTCH1 expression	[246]
compound 29a	GLI protein expression	Medulloblastoma	Inhibition of allograft growth	[247]
Compounds affecting protein regulators of GLI activity				
imiquimod	PKA-mediated GLI phosphorylation	Murine asocellular carcinoma, human medulloblastoma	Downregulation of GLI1 and HHIP expression, GLI3 processing	[248]
forskolin	PKA activation	Pediatric tumors	Inhibition of cell growth, induction of apoptosis	[249]
perifosine	inhibition of GLI1 via AKT/PI3K	Pancreatic cancer	PTCH1 downregulation, cytotoxicity, senzitization to gemcitabine	[250]
		Acute T cell leukemia	Cytotoxycity	[216]
nanoquinacrine	activation of GSK3B	Oral cancer stem cells	Inhibition of cell growth, induction of apoptosis	[251]
Epigenetic regulation of GLI activity				
JQ1	Inhibition of BET bromodomain	medulloblastoma, basocellular carcinoma	Inhibition of cell growth, induction of apoptosis, inhibition of allograft growth	[252]
I-BET151	Inhibition of BET bromodomain	medulloblastoma	Downregulation of GLI1, inhibition of allograft growth	[253]

**Table 3:** Combination therapies targeting GLI proteins in combination with other tumor therapies.

Combination Therapy	Molecular Targets	Model	Effect	Reference
ATO + radiotherapy	GLI + proliferation	High grade neuroepithelial tumor of the central nervous system (primary culture and patient)	Clinical remission for 6 months	[260]
ATO + itraconazole	GLI + SMO	Patients with metastatic basal cell carcinoma	Best overall response was stable disease in 3/5 patients	[261]
GANT61 + obatoclax	GLI + BCL2	Melanoma cells	Decreased cell viability, increased apoptosis	[226]
GANT61 + sunitinib + PF-04691502	GLI + FLT3 + PI3K	Acute myeloid leukemia	Stronger anti-leukemic effects in vivo, prolonged survival of mice	[262]
GANT61 + antiestrogens	GLI + estrogen	ER+ breast cancer cell lines	Decreased cell growth	[263]
GANT61 + metformin	GLI + gluconeogenesis	Prostate cancer cells and xenografts	Decreased cell growth, enhanced radiation response	[264]
GANT61 + paclitaxel	GLI + spindle inhibition	ER+ and triple negative breast cancer	Decreased cell growth in TNBC	[265]
GANT61 + temozolomide	GLI + alkylation/methylation of DNA	Glioma cell lines	Decreased cell growth, increased apoptosis, GANT61 sensitizes cells to TMZ	[266,267]
GANT61 + rapamycin	GLI + mTOR	Pancreatic cancer cell lines and xenografts	Reduced sphere formation and cell viability	[268]
curcumin + resveratrol	HH-GLI signaling	Breast cancer cell lines and xenografts	Induction of apoptosis, 10-fold lower IC_50_ doses in combination compared to individual treatments	[269]
ATO + LY294002	GLI + PI3K	Colon carcinoma cells	Decreased proliferation, synergistic effect	[270]
GANT58 + perifosine	GLI + AKT/PI3K	Acute T cell leukemia	Synergistic cytotoxic effect	[216]
GANT61 + rapamycin	GLI + mTOR	Myeloid leukemia	Growth arrest and apoptosis, synergistic effect	[271]
GANT61 + itraconazole	GLI + antifungal	Breast cancer cell lines	Synergistically enhanced cytotoxicity	[272]
GANT61 + PI103	GLI + PI3K/mTOR	Rhabdomyosarcoma cell lines and xenografts	Synergistic apoptosis induction and tumor growth reduction	[273]
GANT61 + trastuzumab + BEZ235	GLI + ErbB2-PI3K-mTORC1	Esophageal carcinoma	Significantly stronger tumor reduction then individual treatments	[274]
GANT61 + irradiated riboflavin (iRF)	GLI + photosensitizer	Melanoma cells and mouse model	Potentiates the antiproliferative effect of iRF	[275]
GANT61 + cisplatin/doxorubicin/Irinotecan/vincristine	GLI + standard chemotherapy	Neuroblastoma	Synergistic (doxorubicin or vincristine) or additive effects (cisplatin or irinotecan)	[201]
GANT61 + cisplatin	GLI + standard chemotherapy	Biliary tract cancer	Synergistic effect	[212]
GANT61 + rapamycin/temsirolimus	GLI + mTOR	Rhabdomyosarcoma	Reduced survival compared to individual treatments	[220]
ATO + *cis*-platin/ifosfamide/doxorubicin + vismodegib	GLI + standard chemotherapy + SMO	Osteosarcoma	Synergistic effect, inhibition of tumor growth in vivo	[231]
nanoHHI + gemcitabine	GLI + nucleoside synthesis	Pancreatic cancer	Inhibition of tumor growth in vivo	[257]
ATO + cyclopamine	GLI + SMO	Prostate cancer	Synergistic effect, inhibition of tumor growth in vivo	[232]
ATO + LY294002	GLI + PI3K	Colon cancer cells	Synergistic effect	[270]
ATO + gemcitabine	GLI + nucleoside synthesis	Pancreatic cancer cell lines and xenografts	Synergistic effect, inhibition of tumor growth in vivo	[227]

**Table 4:** Resistance mechanisms mediated by GLI in various tumors.

Tumor Type	Resistant to:	Resistance Mechanism	Reference
Lung cancer	Staurosporine	GLI1-mediated upregulation of NDRG1 and downregulation of c-MYC and N-MYC	[276]
	Gefitinib	Upregulation of SHH, SMO and GLI1 (reversible by sulforaphane)	[277]
	Platinum and gefitinib/erlotinib	MEOX-2-dependent GLI1 upregulation	[278]
	Platinum	Hedgehog pathway activation	[279]
	EGFR inhibitors	GLI1-mediated upregulation of SOX2, induction of EMT	[105,280]
Lung and colorectal cancer	Topoisomerase inhibitors	GLI1-mediated upregulation of BID	[281]
Colorectal cancer	5-Fluorouracil	Upregulation of GLI1 and GLI2 and their targets	[282]
	Vorinostat	GLI1-mediated upregulation of BCL2L1	[283]
Gastrointestinal cancer	5-Fluorouracil, cisplatin	GLI2-mediated upregulation of ABCG2 transporter	[284,285]
	Doxorubicin	GLI2-mediated upregulation of ABCG2 transporter	[286]
	Imatinib	GLI-mediated upregulation of KIT	[287]
Esophageal cancer	Chemoradiation	Nuclear localization of GLI1	[288]
Hepatocellular cancer	Sorafenib	GLI2-mediated upregulation of ABCC1 transporter	[289]
Glioblastoma	Temozolomide	GLI1-mediated upregulation of MGMT	[290,291]
Clear cell renal carcinoma	Sunitinib	GLI2 overexpression	[292]
Ovarian cancer	Cisplatin	Rab23-mediated upregulation of ABCG2 through GLI1	[293,294]
Basal cell carcinoma	Gdc-619	SRF-mediated upregulation of GLI1	[295]
Head and neck squamous cancer	Radioresistance	mTOR/SK6-mediated upregulation of GLI1	[296]
Multiple myeloma	Lenalidomide	ADAR1-dependent RNA editing of GLI1	[144]
	Bortezomib	Downregulation of mir-324-5p leading to upregulation of SMO and GLI1	[297]
Chronic myeloid leukemia	Imatinib	GLI1-mediated upregulation of BCR-ABL, p-Akt, Bcl-xl and survivin (reversible by oroxyloside A)	[298]
Acute myeloid leukemia	Radiation	Activation of the GLI1/PI3K/AKT/NFKB pathway	[299]
	Ribavirin/cytarabine	GLI1-mediated upregulation of UDP-glucuronosyl transferase enzymes	[300]
